# Obstructive Sleep Apnea Is Associated with Elevated High Sensitivity C-Reactive Protein Levels Independent of Obesity: Korean Genome and Epidemiology Study

**DOI:** 10.1371/journal.pone.0163017

**Published:** 2016-09-29

**Authors:** Jinkwan Kim, Seok Jun Lee, Kyung-Mee Choi, Seung Ku Lee, Dae Wui Yoon, Seung Gwan Lee, Chol Shin

**Affiliations:** 1 Department of Biomedical Laboratory Science, College of Health Science, Jungwon University, Chung-Buk, Republic of Korea; 2 Department of Biomedical Laboratory Science, College of Health Science, Cheong-Ju University, Chung-Buk, Republic of Korea; 3 Institute of Human Genomic Study, Korea University Ansan Hospital, Korea University, Ansan, Republic of Korea; 4 Department of Pulmonary Sleep and Critical Care Medicine Disorder Center, College of Medicine, Korea University, Ansan, Republic of Korea; 5 Department of Health and Integrative Science, College of Health Science, Korea University, Seoul, Republic of Korea; University of Rome Tor Vergata, ITALY

## Abstract

Obstructive sleep apnea syndrome (OSA) has been recognized as a common health problem, and increasing obesity rates have led to further remarkable increases in the prevalence of OSA, along with more prominent cardiovascular morbidities. Though previous studies have reported an independent relationship between elevated high sensitivity C-reactive protein (hsCRP) levels and OSA, the issue remains controversial owing to inadequate consideration of obesity and various confounding factors. So far, few population based studies of association between OSA and hsCRP levels have been published. Therefore, the purpose of the present study was to investigate whether OSA is associated with increased hsCRP levels independent of obesity in a large population-based study. A total of 1,835 subjects (968 men and 867 women) were selected from a larger cohort of the ongoing Korean Genome and Epidemiology Study (KoGES). Overnight polysomnography was performed on each participant. All participants underwent anthropometric measurements and biochemical analyses, including analysis of lipid profiles and hsCRP levels. Based on anthropometric data, body mass index (BMI) and waist hip ratio (WHR) were calculated and fat mass (FM) were measured by means of multi-frequency bioelectrical impedance analysis (BIA). Mild OSA and moderate to severe OSA were defined by an AHI >5 and ≥15, respectively. The population was sub-divided into 3 groups based on the tertile cut-points for the distribution of hsCRP levels. The percentage of participants in the highest tertile of hsCRP increased dose-dependently according to the severity of OSA. After adjustment for potential confounders and obesity-related variables (BMI, WHR, and body fat) in a multiple logistic model, participants with moderate to severe OSA had 1.73-, 2.01-, and 1.61-fold greater risks of being in the highest tertile of hsCRP levels than participants with non-OSA, respectively. Interaction between obesity (BMI ≥25kg/m^2^) and the presence of moderate-to-severe OSA was significant on the middle tertile levels of hsCRP (OR = 2.4), but not on the highest tertile, compared to the lowest tertile. OSA is independently associated with elevated hsCRP levels and may reflect an increased risk for cardiovascular morbidity. However, we found that OSA and obesity interactively contribute to individuals with general levels of hsCRP (<1.01 mg/dl). The short-term and long-term effects of elevated hsCRP levels on cardiovascular risk in the context of OSA remain to be defined in future studies.

## Introduction

Obstructive Sleep Apnea (OSA) is caused by repetitive obstruction of the upper airway during sleep, which results in an increase in resistance in the upper airway, leading to snoring and repetitive occurrences of intermittent hypoxia and hypercapnia in the body; thus, cyclical frequent arousals cause sleep fragmentation. Increasing evidence from several lines of investigation strongly supports the concept that OSA is pathophysiologically linked to cardiovascular diseases (CVD) such as hypertension, ischemic heart disease, and cerebrovascular disease [1, 2]. Increased generation of reactive oxygen species and systemic inflammatory responses related to hypoxia-reoxygenation events and to sleep fragmentation are mechanistically involved in the acceleration and propagation of atherogenesis [3, 4]. However, the mechanisms underlying the association between OSA and CVD are currently not fully understood. Furthermore, given that the prevalence of obesity, which is known to be a strong risk factor for OSA, is rapidly rising in Asian countries, the substantial attention to OSA-related comorbidities is expected to continue increasing.

C-reactive protein (CRP), which is known to be an important biomarker of CVD, is an acute-phase protein that is generated in the liver by IL-6. CRP is more specifically found in the lesions of atherosclerosis and is thought to participate actively in atheromatous lesion formation through the induction and enhanced expression of adhesion molecules [5]. Increased levels of CRP have been found in both adults [6–8] and children with OSA [9–12], and CRP levels are substantially reduced after treatment [8, 11, 13]. However, not all studies in adults [14, 15] or in children [16, 17] have confirmed the putative association between CRP levels and OSA severity, owing to small sample sizes and inadequate consideration of confounding factors such as obesity. Moreover, a few population-based studies reported an association between OSA and CRP [18]. Therefore, we sought to investigate whether OSA is associated with elevated hsCRP levels independent of obesity in a large population-based study, and to evaluate the significance of hsCRP as a biomarker that can be used to help predict and reduce the risk of CVD in the context of OSA.

## Subjects and Methods

### Subjects

Participants in the present study were part of a larger study, the Korean Genome and Epidemiology Study (KoGES), which is an ongoing, population-based cohort study that started in 2001 under the original title “The Korean Health and Genome Study.” Detailed information on the study design and aims of the KoGES has been reported previously [19–21]. In brief, the original study was designed to establish a representative adult cohort in the city of Ansan, Korea, and to identify the epidemiologic characteristics, frequencies and determinants of chronic diseases among Koreans. From June 2001 to January 2003, a longitudinal cohort was formed, consisting of 5,015 participants (2,521 men and 2,494 women, 40–69 years of age) who participated in a comprehensive health examination and on-site interviews at Korea University Ansan Hospital. Follow-up assessments were conducted biennially with scheduled site visits. At each visit, participants signed an informed consent form and this study was approved by the Human Subjects Review Committee at Korea University Ansan Hospital. Polysomnography (PSG) was included in the study protocol in September 2009 in about half of the KoGES participants. Although PSG will eventually be administered to the entire study population, subjects for the present study include only those with PSG data from the fourth biennial examination from March 2007 to February 2009. After excluding participants who had missing data and those with extreme outliers of biochemical data, a total of 1835 individuals (968 men and 867 women) were recruited into the current study. Participants were also excluded if they had any known systemic inflammatory disease or genetic abnormality using a questionnaire for a general health examination or had a high hsCRP level (hsCRP level >10mg/dl) or, had received any treatment for OSA. All participants underwent anthropometric measurements, a general health examination, an assessment of sleep habits, and polysomnography (PSG).

### Overnight polysomnography (PSG)

An overnight sleep study was performed at each participant’s home with a portable device (Embletta® X-100; Embla Systems, San Carlos, CA, USA), as previously described [22]. In brief, two trained sleep technologists visited participants’ homes in the evening, applied sensors, and instructed participants on how to turn the sensors on and off. Participants also were required to record the times they turned the lights on and off and report those times the following morning. The recording channels were as follows: one electroencephalography (C4-A1), one electrooculography (right upper outer canthus-left lower outer canthus), one chin electromyography, one modified lead II electrocardiography, one airflow from nasal airflow pressure transducer, two respiratory effort from chest and abdominal respiratory inductance plethysmography, one pulse oximeter, and one position sensor. For qualified data, sleep status and respiratory events were scored according to standard guidelines. Obstructive apnea was defined as a clear decrease (≥90%) from baseline in the amplitude of the nasal pressure with ongoing chest and abdominal movement, and hypopneas were identified if there was ≥30% reduction in the nasal pressure from baseline, associated with at least 4% oxygen desaturation on pulse oximetry. The duration threshold for these respiratory events was 10 sec. Mild OSA and moderate-to-severe OSA were defined by an AHI >5 and ≥15, respectively. Arousals were defined according to the American Academy of Sleep Medicine Scoring Manual [23, 24].

### Anthropometrics, biochemical data, and assessment of body composition

All subjects were surveyed for their age, sex, marital status, smoking, drinking, and other habits. Blood pressure, height, weight, waist circumference and hip circumference were measured according to standard protocols, as previously reported [22]. Body mass index (BMI) was calculated as the weight divided by the square of height. Obesity was defined with BMI ≥25kg/m^2^, according to the Asian-specific BMI cut-off from the World Health Organization Report [25]. For biochemical tests, when participants visited for their follow-up examinations, blood was collected after the subjects had fasted for at least 8 hours. Biochemical data including glucose, HDL, LDL, triglyceride, and hsCRP levels were measured (ADVIA 1650 and 1680, Siemens, Tarrytown, NY, USA). Body composition was measured by means of multi-frequency bioelectrical impedance analysis (BIA) with 8-point tactile electrodes (InBody 720; Biospace, Seoul, Korea) [26, 27]. This analyzer uses an alternating current of 250 mA at variable frequencies of 1, 5, 50, 250, 500, and 1,000 kHz. Fat-free mass and fat mass were obtained from a multi-frequency BIA.

### Statistical analysis

Data are expressed as mean±SD. Significant association of hsCRP with continuous and categorical variables were identified by ANOVA and Chi-square test, respectively. Bonferroni correction was applied for multiple comparisons. The population was grouped based on tertile cut-points of hsCRP levels (>1.01 mg/dl for the highest tertile and <0.42 mg/dl for the lowest tertile). Then, univariate and stepwise multivariate linear regression analyses were done for hsCRP with explained by OSA, obesity, and other variables. Using a logistic regression, odds ratio and 95% confidence interval were estimated for the highest tertile of hsCRP levels compared to the lowest tertile. Statistical significance was identified at the 0.05 level. All the statistical analyses were done using SPSS software (version 18.0; SPPS Inc., Chicago, IL, USA).

## Results

### Study population

The demographic, polysomnographic, and biochemical characteristics of the 3 groups, divided by the severity of the AHI, are presented in Table 1. Regarding the presence or absence of OSA, 611 participants had mild OSA, 251 had moderate-to-severe OSA, and 973 were non-OSA. The mean ages of participants in the 3 groups were 58, 57 and 53 years, respectively. Polysomnographic data, including the AHI and the SaO2 nadir, showed significant group differences (p<0.01). Metabolic profiles, including those for glucose, triglyceride and HDL cholesterol levels, differed significantly among the 3 groups (p<0.01); however, total cholesterol did not vary significantly. HsCRP levels increased dose-dependently according to the severity of OSA (Moderate-to-Severe OSA vs. Mild OSA vs. Non-OSA among non-obese participants, 1.47±1.60 mg/dL vs. 1.20±1.34 mg/dL vs. 0.97±1.22 mg/dL, p<0.01).

**Table 1:** General Characteristics of Study Participants.

	Moderate-to-severe OSA (AHI>15)	Mild OSA (5<AHI≤15)	Non-OSA (AHI<5)	P value^1^^)^
N (%)	251 (13.7)	611 (33.3)	973 (53.0)	-
Age (years)	58.0±7.7	57.2±7.2‡	53.8±6.6&	<0.01
Male, n (%)	167 (66.5)	348 (57.0)	453 (46.6)	<0.001
BMI (kg/m2)	25.9±3.2†, §	25.1±2.6‡	23.9±2.6	<0.01
WHR (waist/hip)	0.89±0.06†, §	0.87±0.06‡	0.84±0.06	<0.01
FM/Body Weight (kg/kg)	0.27±0.06§	0.27±0.06‡	0.26±0.06	<0.01
FFM/Body Weight (kg/kg)	0.72±0.06§	0.73±0.06‡	0.74±0.06	<0.01
ESS	5.89±4.14	5.75±4.57	5.85±4.36	0.868
Current Smoker, n (%)	130 (51.8)	253 (41.4)	350 (36.0)	<0.01
Current Drinker, n (%)	166 (66.1)	356 (58.3)	504 (51.8)	<0.01
Medication for HTN, n (%)	71 (34.6)	159 (30.3)	39 (20.6)	<0.01
Medication for DM, n (%)	30 (14.6)	67 (12.8)	16 (8.5)	<0.01
Systolic BP (mmHg)	117.1±14.3†	113.3±13.4‡	109.6 ±13.8	<0.01
Diastolic BP (mmHg)	77.7 ±9.8†	75.5 ±9.2‡	73.3 ±9.4	<0.01
AHI (events/hour)	24.7 ±10.8†	8.8 ±2.7‡	1.9 ±1.4	<0.01
(Median, IQR)	(21.3, 17.3–28.6)	(8.6, 6.4–10.9)	(1.7, 0.7–3.2)	
SaO2 Nadir (%)	81.4 ±5.3†	85.4±3.8‡	90.2 ±2.9	<0.01
(Median, IQR)	(82.0, 79.0–85.0)	(86.0, 83.0–88.0)	(91.0, 89.0–92.0)	
Fasting Glucose (mg/dL)	107.9 ±40.4†	101.5±32.0‡	96.7 ±28.5	<0.01
Total Cholesterol (mg/dL)	197.4 ±36.5	201.7±34.1	199.7 ±34.7	0.221
HDL cholesterol (mg/dL)	42.8 ±9.2	44.1 ±10.6‡	45.6 ±10.9&	<0.01
Triglyceride (mg/dL)	161.1 ±93.2†	147.7 ±95.0§	126.8±76.9&	<0.01
HsCRP (mg/dL)	1.47±1.60†	1.20±1.34‡	0.97±1.22&	<0.01
(Log-transformed)	(-0.03±0.43)	(-0.10±0.39)	(-0.22±0.42)	

All data are expressed as mean±SD. Statistical significance was estimated after logarithmic transformation if the data were not normally distributed. BMI, body mass index; WHR, waist hip ratio, FM, fat mass, FFM, free fat mass, ESS, Epworth sleepiness scale; HTN, hypertension; DM, diabetes; BP, blood pressure; AHI, apnea hypopnea index; IQR, interquartile range; HDL, high-density lipoprotein; hsCRP, high sensitivity C-reactive protein.

^1)^ The combinatory association is significant for P-value<0.05.

†P<0.01, Mild OSA vs. Moderate to severe OSA

‡P<0.01, Mild OSA vs. Non-OSA

^§^P<0.05, Mild OSA vs. Moderate to severe OSA

^&^P<0.01, Moderate to severe OSA vs. Non-OSA

### Percentage of participants in the highest tertile of hsCRP levels according to the severity of OSA, based on the presence of obesity

**Fig 1:**
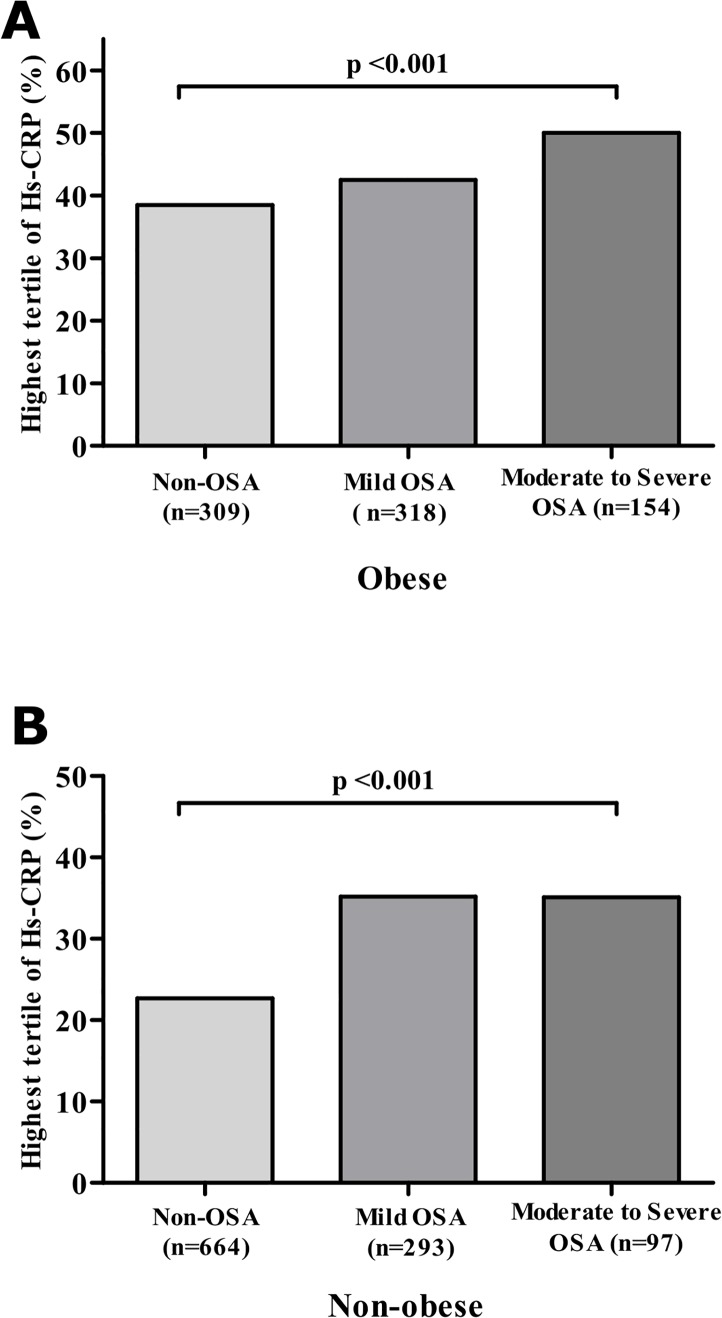
Percentage of participants in the highest tertile of hsCRP levels according to the severity of OSA in the obese and non-obese groups. Obesity was defined by a BMI ≥25 kg/m^2^ according to the Asian-specific BMI cut-offs from the World Health Organization Report. A) Percentage of participants in the highest tertile of hsCRP levels among 3 groups of obese participants. B) Percentage of participants in the highest tertile of hsCRP levels among 3 groups of non-obese participants.

Fig 1 shows the percentage of participants in the highest tertile of hsCRP levels according to the severity of OSA by the presence or absence of obesity. Because obesity was expected to contribute to increased hsCRP levels as a major confounding factor, we compared the percentage in the highest tertile among the 3 groups of both obese and non-obese participants. Even after controlling for obesity (BMI≥25 kg/m^2^), we found that the portion of participants in the highest tertile of hsCRP levels differed significantly according to the severity of OSA (Fig 1A, Moderate-to-Severe OSA vs. Mild OSA vs. Non-OSA among obese participants, 50.0% vs. 42.5% vs. 38.5%, p<0.001; Fig 1B, Moderate-to-Severe OSA vs. Mild OSA vs. Non-OSA among non-obese participants, 35.1% vs. 35.2% vs. 22.7%, p<0.001). Besides, significant differences in hsCRP levels among the 3 groups were exhibited in both the obese and non-obese groups (Moderate-to-Severe OSA vs. Mild OSA vs. Non-OSA among obese participants, 1.69±1.73 mg/dL vs. 1.29±1.35 mg/dL vs. 1.20±1.29 mg/dL, p<0.01; Moderate-to-Severe OSA vs. Mild OSA vs. Non-OSA among non-obese participants, 1.12±1.31 mg/dL vs. 1.11±1.32 mg/dL vs. 0.87±1.17 mg/dL, p<0.01).

### Association between the severity of OSA and hsCRP in participants

To examine independent predictors of hsCRP levels in participants, we performed regression analyses (Table 2). In univariate analysis, hsCRP level was positively associated with AHI (n = 1835, β±SE; 0.126±0.02, p<0.01). In multiple regression analysis, hsCRP level was positively associated with AHI (β±SE; 0.055 ±0.02, p<0.01; Table 2) and negatively associated with the SaO2 nadir (β±SE; -1.33±0.41, p<0.01) after adjustment for potential confounding factors including age, sex, smoking status, alcohol use, DM medication, HTN medication, and BMI. In order to further estimate the odds ratio having a given hsCRP level based on the presence and severity of OSA, we performed logistic regression analysis. Table 3 presents the univariate and multivariate odds ratios of being in the highest versus the lowest tertile of hsCRP levels, according to the severity of OSA. In the univariate model, the odds ratios of being in the highest tertile of hsCRP (>1.01 mg/mL) versus the lowest tertile (<0.42 mg/mL) were 2.27 (95% CI, 1.76–2.93, p<0.01) and 2.91 (95% CI, 2.04–4.16, p<0.05) for subjects with mild OSA (5<AHI≤15) and moderate-to-severe OSA (AHI>15), respectively, with reference to non-OSA subjects. After adjustment for age, sex, smoking status, alcohol use, DM medication, HTN medication, and each of the obesity-related variables (BMI, WHR, and FM/body weight), participants with moderate-to-severe OSA had 1.73- (BMI adjusted), 2.01- (WHR adjusted), and 1.61- (FM/body weight adjusted) fold greater risks (95% CI, 1.14–2.62, p<0.05) than non-OSA subjects of being in the highest tertile of hsCRP compared with the lowest tertile. Interestingly, participants with moderate-to-severe OSA had the highest odds ratio for being in the highest tertile of hsCRP levels compared with non-OSA participants after adjustment for confounding factors and WHR, which is major predictor of OSA.

**Table 2:** Multivariate linear regression analyses between AHI or SaO2 nadir and hsCRP levels after adjustment for confounding factors.

	HsCRP levels†											
	Crude			Model 1*			Model 2**			Model 3***		
Independent Variables	Beta	SE	P-value	Beta	SE	P-value	Beta	SE	P-value	Beta	SE	P-value
AHI†	0.126	0.018	<0.01	0.055	0.02	<0.01	0.078	0.019	<0.001	0.066	0.019	<0.001
SaO2 nadir†	-2.892	0.386	<0.01	-1.33	0.41	<0.01	-1.885	0.402	<0.001	-1.608	0.406	<0.001

Abbreviation: AHI, apnea hypopnea index; SE, standard error

† Data were log-transformed

*Adjusted for confounding factors including age, sex, smoking status, alcohol use, DM medication, HTN medication, and BMI.

**Adjusted for confounding factors including age, sex, smoking status, alcohol use, DM medication, HTN medication, and WHR.

***Adjusted for confounding factors including age, sex, smoking status, alcohol use, DM medication, HTN medication, and FM/body weight.

**Table 3:** Estimated odds ratio of being in the highest tertile of hsCRP levels according to the severity of OSA.

	Sample Size (n)	Estimated Odds Ratio (95% CI)							
		Highest Tertile vs. Lowest Tertile of HsCRP Levels							
		Crude	P-value	Model 1*	P-value	Model 2**	P-value	Model 3***	P-value
Non-OSA (AHI<5)	733	Reference	-	Reference	-	Reference	-	Reference	-
Mild OSA (5<AHI≤15)	189	2.27 (1.76–2.93)	<0.01	1.58 (1.17–2.12)	<0.01	1.78 (1.33–2.39)	<0.01	1.55 (1.33–2.39)	<0.01
Moderate-to-severe OSA (AHI>15)	205	2.91 (2.04–4.16)	<0.01	1.73 (1.14–2.62)	<0.05	2.01 (1.33–3.04)	<0.01	1.61 (1.06–2.46)	<0.05
Mild OSA vs. Moderate to Severe OSA	-	2.27 (1.76–2.93)	<0.01	1.57 (1.16–2.12)	<0.01	1.75 (1.30–2.36)	<0.01	1.65 (1.23–2.23)	<0.01

Logistic regression analysis was used to estimate odds ratios and 95% confidence intervals after the cohort was divided into 3 groups based on tertile cut-points according to the distribution of hsCRP levels for the whole cohort.

*Adjusted for confounding factors including age, sex, smoking status, alcohol use, DM medication, HTN medication, and BMI.

**Adjusted for confounding factors including age, sex, smoking status, alcohol use, DM medication, HTN medication, and WHR.

***Adjusted for confounding factors including age, sex, smoking status, alcohol use, DM medication, HTN medication, and FM/body weight.

### Interaction between OSA and obesity on hsCRP level

After the tertile stratification of hsCRP, log-transformed hsCRP mean was compared among the two OSA groups and non-OSA group using ANOVA with Bonferroni post-hoc analysis. There was a significant difference between moderate-to-severe OSA and mild OSA (mean ± standard error, 0.086 ±0.033), but only for the group of highest hsCRP tertile with obesity (BMI ≥25 kg/m^2^). Lower tertile groups were not significantly different depending on OSA, regardless of obesity. Further to identify interaction between obesity and OSA on hsCRP, odds ratios of the highest and middle tertiles hsCRP were estimated, compared to the lowest tertile, respectively, using a logistic regression model explained by age, sex, smoking, drinking, hypertension and diabetes medications, obesity, OSA, and interaction of obesity and OSA. As the result, a significant interaction between obesity and the presence of OSA was observed for the middle tertile group of hsCRP only (adjusted odds ratio [OR], 2.4; 95% CI, 1.1–5.4, p = 0.030). In the highest tertile group of hsCRP, the interaction was not significant, but obesity (OR, 1.9; 95% CI, 1.3–3.6, p = 0.001) and mild OSA (OR, 1.9; 95% CI, 1.3–2.8, p = 0.003), diabetes medication (OR, 1.8; 95% CI, 1.2–2.8, p = 0.003) and age (OR, 1.02; 95% CI, 1.01–1.04; p = 0.013) were all significant. Additionally, without the tertile classification of hsCRP, the interaction between obesity related variables BMI, WHR, and OSA on hsCRP was examined using a generalized linear model, in which no such a significant interaction was identified.

## Discussion

In this large population-based study, we found that hsCRP levels increased dose-dependently according to the severity of OSA, even in non-obese participants. Furthermore, the percentage of participants in the highest tertile of hsCRP levels increased dose-dependently according to the severity of OSA, independent of obesity. In multiple regression analysis, hsCRP levels were positively associated with the AHI and negatively associated with the SaO2 nadir, even after adjustment for potential confounding factors and each of the three obesity-related variables (BMI, WHR, and FM). Moreover, in an attempt to further estimate the odds ratio of having a given hsCRP level based on the presence of OSA, we also performed logistic regression analysis. Even after adjustment for various confounding factors and each of the obesity-related variables, participants with moderate-to-severe OSA had 1.78- (BMI adjusted), 2.01- (WHR adjusted), and 1.61-fold (Fat mass/body weight) greater risks of being in the highest tertile of hsCRP levels than non-OSA participants (Table 3), supporting the hypothesis that OSA is associated with elevated levels of hsCRP. However, we also found a significant interaction between obesity and the presence of OSA in the middle tertile group of hsCRP only. Thus, we suggest that other additional confirmatory studies should address the issue of whether obesity level estimated by the different indexes influences how OSA affects hsCRP level in a large population based study more detail.

HsCRP, a ubiquitous protein that can be generated in multiple cell types, is a robust biomarker of underlying systemic inflammation and is regulated by the proinflammatory cytokines, particularly IL-6 and TNF-α [3]. Although the exact mechanisms linking OSA to the inflammatory cascade are not clear, and remain to be fully elucidated, the intermittent hypoxia and reoxygenation that characterize OSA contribute to the cumulative burden of oxidative stress and the generation of reactive oxygen species (ROS), and trigger inflammatory cytokines[4, 28]. In the last two decades, the CRP level has been extensively assessed as an independent marker of future cardiovascular events and metabolic dysfunction [29–32], in addition to endothelial function and integrity. Recent studies revealed that low grade inflammation is reflected by increased levels of hsCRP in patients with type 2 diabetes and small increased CRP level predict the likelihood of developing cardiovascular events both in diabetic and nondiabetic populations [33–35]. Not surprisingly, previous studies have reported a strong association between OSA and hsCRP levels that is independent of other well-established risk factors. After the association between OSA and hsCRP levels was reported initially by Shamsuzzaman and colleagues [36], many other investigators confirmed that OSA is associated with elevated hsCRP levels, and that such increases in hsCRP levels are reversed by effective treatment of the underlying sleep-disordered breathing [37–51]. Yokoe and colleagues [37] reported that both the level of CRP and the spontaneous production of IL-6 by monocytes were elevated in patients with OSA. Guven and colleagues [52] also found that OSA was associated with elevated CRP levels independent of obesity. Moreover, Lui et al. revealed that elevated CRP levels were associated with OSA independent of visceral obesity in healthy middle-aged men [45]. However, in most of these studies, a case-control study design was used, and a small number of patients suspected of having OSA were studied in clinical settings. More substantial data from a community-based study demonstrated that nocturnal intermittent hypoxia was associated with elevated CRP levels among middle-aged Japanese subjects [48].

However, even though incremental data from both clinical and epidemiological studies have demonstrated a positive association between elevated CRP and OSA, the issue is still controversial. A number of other studies did not find this relation, as these studies did not adequately consider analytical confounders, such as central obesity, smoking status, or hypertension. In a cross-sectional study to determine whether obesity or OSA is responsible for increased levels of CRP in patients with sleep disordered breathing (SDB), Sharma et al. found that obesity, and not obstructive sleep apnea, was associated with elevated serum levels of hsCRP [53]. Guilleminault et al. reported that CRP levels were significantly correlated with BMI and esophageal pressure, and only BMI was significantly associated with high CRP values [15]. Moreover, Taheri et al. found no independent relationship between CRP levels and indices of SDB in 907 adults enrolled in the Wisconsin Sleep Cohort who had undergone inpatient PSG, suggesting that the relationship between these two variables may be driven primarily by their association with obesity [54]. Recently, the Icelandic Sleep Apnea Cohort study interestingly revealed that OSA severity was an independent predictor of IL-6 and CRP levels, but interacted with obesity such that this association was found only in obese patients [55]. In the present study, because obesity was expected to contribute to increased hsCRP levels, we repeated our analysis to examine the percentage of subjects in the highest hsCRP tertile among the 3 groups by severity of OSA after adjusting for obesity. In the analysis stratified by obesity, the percentage of subjects in the highest hsCRP tertile increased according to the severity of OSA in both the obese and non-obese groups. Moreover, in an attempt to examine whether OSA is responsible for increases in hsCRP levels independent of obesity, we separately tested three obesity-related variables (BMI, WHR, and FM/body weight) in regression models. These variables are commonly useful indices of obesity and central obesity in public health and population-based studies. Even though there was a significant interaction between obesity and the presence of OSA in the middle tertile group of hsCRP only, but this was not significant in the highest tertile group of hsCRP (>1.01 mg/dl). This finding indicates that obesity is not fully affect in a wide ranged hsCRP level in OSA. Recently, obesity has become one of the social problems in Korea, and the major cause of the increasing obesity is known to be the change of lifestyle and nutrition. Several studies demonstrated that Asians show markedly different etiology in the onset of obesity-related morbidities [56] and they may have a different susceptibility to OSA [57] and a different pro-inflammatory profile to Caucasians and African Americans [58]. Obviously, since obesity and OSA commonly coexist, more specific assessment of these factors in the context of OSA will have to investigate in future studies.

Compared with previous studies, many of which had methodological limitations, the current study has several strengths. The strengths are the large sample size and community-based study design in the general population among subjects with relatively low BMIs. Most of the previous studies were performed under the constraints of clinical practice and failed to adequately exclude confounding factors while addressing the issue. A precise estimation of the actual role of obesity is essential if the analysis of hsCRP levels is to be devoid of known confounders. In the present study, we included study participants who were well within the normal range of BMIs in the Asian population (Mean±SD, 24.6±2.85). Thus, our results could be representative of the general population. Another advantage of the present study was the evaluation of OSA with a portable PSG at home, which provided a more realistic assessment of OSA severity than can be achieved in clinical-based studies by allowing the maintenance of regular daily habits of sleep, physical activity, and diet in the general population.

While the present study included a large general population sample and extensive adjustment for obesity with various variables, several limitations should be addressed. First, we did not consider the influence of genetic variance and environmental factors, which play an important role in regulating hsCRP levels. Previous studies have shown that genetic variation in the IL-6/CRP pathway is associated with increased risk for OSA, suggesting that it may account for the higher CRP levels in the context of OSA [59, 60]. It will definitely be important to explore the impact of specific CRP gene polymorphisms on these associations. Second, we did not elucidate the possibility of reverse causality—for instance, whether any treatments for OSA, such as CPAP treatment or surgical treatments, could reduce hsCRP levels. Even though significant reduction in hsCRP levels after CPAP treatment has been reported previously, this issue remains controversial [61]. Therefore, well-designed, randomized controlled trials are needed to address this issue in future research. Third, our results from separately controlling for each of obesity-related factors such as BMI, WHR, and FM in regression model may not sufficient to exclude the influence of obesity on the association between OSA and elevated CRP levels. A previous study from the Asian patients demonstrated an independent associations of AHI and CRP levels, adjusting for BMI and visceral fat measured by MRI [45]. However, MRI measurement of visceral fat mass is an expensive and laborious technique, so we did not use this technique to estimate visceral obesity in the present study population. It definitely will be important to use highly advanced techniques for evaluating obesity to further explore the role of fat distribution in the elevation of hsCRP levels in OSA. Fourth, another potential limitation is that the blood sample for assessment of hsCRP level was not immediately drawn the morning after PSG study. Our data showed that the mean of time difference between dates for performing PSG and blood collection from the participants was 55.3 days. Thus, this difference may be influence of estimating association between hsCRP level and OSA. Finally, we could not consider the effect of alterations of glucose metabolism or insulin resistance, which may also play an important role in elevation of hsCRP level in OSA. Since several studies revealed that significant associations between severity of OSA, insulin resistance, and BMI was found [62, 63]. Moreover, we would like to emphasize that the present study was cross-sectional; thus, the findings of significant associations are insufficient to infer causality. Therefore, well-designed, prospective cohort studies are needed to address whether alterations of glucose and insulin level as well as obesity are interactively associated with elevated hsCRP level in the context of OSA in the future.

In summary, elevated hsCRP levels are independently associated with OSA and may reflect increased risk for cardiovascular morbidity. However, we found that OSA and obesity interactively contribute to elevation of hsCRP levels (middle ranged). Obviously, more specific assessment of interaction between obesity and OSA on elevation of hsCRP level will have to investigate and the short-term and long-term effects of elevated hsCRP levels on cardiovascular risk in the context of OSA remain to be defined in future studies.
